# Use of positive expiratory pressure during six minute walk test: results in patients with moderate to severe chronic obstructive pulmonary disease

**DOI:** 10.1186/2049-6958-8-19

**Published:** 2013-03-14

**Authors:** Antonello Nicolini, Federico Merliak, Cornelius Barlascini

**Affiliations:** 1 Respiratory Diseases Unit,Hospital of Sestri Levante, Via Terzi 43-16039, SestriLevante, Italy; 2 Forensic Medicine, ASL4 Chiavarese, Italy

**Keywords:** Moderate-severe chronic obstructive pulmonary disease, Positive expiratory pressure device, Six-minute walk test

## Abstract

**Background:**

The six-minute walk test (6MWT) is widely used because it is both simple and reliable as a measure of exercise capacity. Individuals with chronic obstructive pulmonary disease (COPD) usually show a limited capacity to perform exercise.

**Methods:**

Our study is a prospective, randomized controlled trial which uses the 6MWT in one hundred consecutive in and out- patients with moderate to severe COPD to assess the benefit of a simple positive expiratory pressure (PEP) device. PEP device consisted of a PEP valve 5 cmH_2_O connected to 1-meter tube and a mouthpiece. All the enrolled patients performed a 6MWT before randomization. The following day PEP group patients performed the 6MWT using PEP device. Control group patients performed the 6MWT without this device. The primary outcome was the difference in distance (meters) walked.

**Results:**

Functional capacity assessed by the distance covered during 6MWT improved in the PEP group more than in the control group. The difference was statistically significant (p < 0.001).Oxygen saturation improved to a statistically significant level during 6MWT (p < 0.01). Heart rate was also reduced (p < 0.03).

**Conclusions:**

There are few studies demonstrating that PEP devices enhance exercise capacity in COPD patients. Our results has been obtained using only a low positive expiratory pressure (5 cmH_2_O). In our opinion the strength of this study is the simplicity and the lower cost when compared to other devices and approaches. The study was registered as Chi CTR-ORC-12002173 at http://www.chictr.org.

## Background

Exercise capacity is the most common measure of cardiovascular and metabolic efficiency. The six minute walk test (6MWT) is a standard procedure used to evaluate exercise capacity in pulmonary and cardiac patients [1]. Although the cardiopulmonary exercise test with gas exchange is the “gold standard” method for measuring exercise in respiratory medicine, its use in routine clinical practice is limited because it requires expensive and complex technology. The 6MWT is the most widely used because it is both simple and reliable as a measure of exercise capacity.

The 6MWT is internationally used to measure functional status and prognosis in patients with a wide variety of diseases, such as pulmonary hypertension, congestive heart failure or chronic obstructive pulmonary disease (COPD). It can also be utilized to investigate the effects of several interventions (rehabilitation, pharmacological therapy, oxygen supplementation) on the patients’ walking capacity [2,3]. This test, referred to as a sub-maximal high-intensity constant-load exercise, is conducted for a period of 6 minutes while the patient is walking as far as possible. A supervised measurement of the distance walked (in meters), with associated pulse oximetry evaluations, and rating of dyspnea detected with the Borg scale or with visual analogic scale are then recorded as outcome measures [3]. In COPD patients the distance walked correlates moderately with either an individual’s health-related quality of life, symptoms, peak work capacity as assessed by CPET or pulmonary function [2,3]. Most important, the distance walked is a surrogate marker of long-term survival in COPD patients, even when they are in the most advanced status of their disease [3].

Many patients with chronic respiratory diseases, particularly those with COPD present a dysfunction related to a disorder of the skeletal muscles that reflects a limitation in exercise capacity [3]. COPD is a pulmonary disorder that is characterized by progressive irreversible airflow limitation resulting from alveolar wall destruction, bronchiolar narrowing, and airways inflammation [4]. Individuals with COPD usually show a limited capacity to perform exercise. When compared to healthy individuals they demonstrate lower maximum exercise capacities with the lowest levels observed in subjects with more severe COPD [5]. Patients with COPD typically experience dyspnea during exercise and stop exercising because of dyspnea or leg fatigue or a combination of both. Patients with mild COPD usually perceive dyspnea more intensely than leg fatigue [6,7]. In this group the breathing pattern is more rapid and shallow and is the cause of dynamic hyperinflation which generates an inspiratory threshold due to positive end-expiratory pressure which results in a reduction of inspiratory capacity strictly related to dyspnea and in respiratory effort [7]. Dynamic hyperinflation is the result of expiratory flow limitation: most people with COPD are able to maintain a stable end expiratory lung volume (EELV) and inspiratory capacity (IC) at rest. However, with the increased ventilatory demand imposed by exercise, the expiratory flow limitation arise. This leads to increased EELV and reduced IC.These two parameters have been identified as major contributory factors to dispnea in COPD patients [8].Treatments to reduce airflow obstruction and/or dynamic hyperinflation include pursed lip breathing (PLB) [9], several non-pharmacological therapies that include supplemental oxygen, heliox, breathing helium-oxygen mixtures, and non-invasive ventilation [7,9]. There is evidence that non-invasive ventilation reduces the work of breathing during exercise which in turn decreases dyspnea and increases endurance time in patients with moderate to severe COPD. Several studies have demonstrated a relationship between decreased dyspnea and reduced work of breathing [7,10-12]. Only few evidences exist concerning the use of positive expiratory devices: these devices should permit a reduction in lung hyperinflation and an increasing in exercise duration (endurance) [13]. On this regard we decided to investigate if the use of a positive expiratory pressure device could improve the distance walked by patients with moderate to severe COPD.

## Methods

### Patients and study design

A prospective, randomized controlled trial was performed from April 2012 to September 2012 in consecutive in-and outpatients with moderate to severe COPD recruited at the Respiratory Diseases Unit of the General Hospital of Sestri Levante (GE, Italy). Inclusion criteria were: age from 18 to 80 years, and clinical stability (no change in medication within a week prior to the test). Exclusion criteria were: history of bronchial asthma, inability to perform the six minute walk test, or absence of written consent.

A randomization plan was generated by a statistician not involved in the study using a randomization table from a computer software program. The randomization assignments were provided to the recruiting physician in sealed envelopes. The investigators who performed the study’s data analysis were blinded to the patients’ assignments.

The study was carried out in accordance with the Helsinki Declaration. All patients provided written informed consent before beginning the study. The study was registered as Chi CTR-ORC-12002173 at http://www.chictr.org.

### Protocol

We assessed the effects of a positive expiratory pressure (PEP) device during 6MWT in patients with moderate to severe COPD.

**Figure 1:**
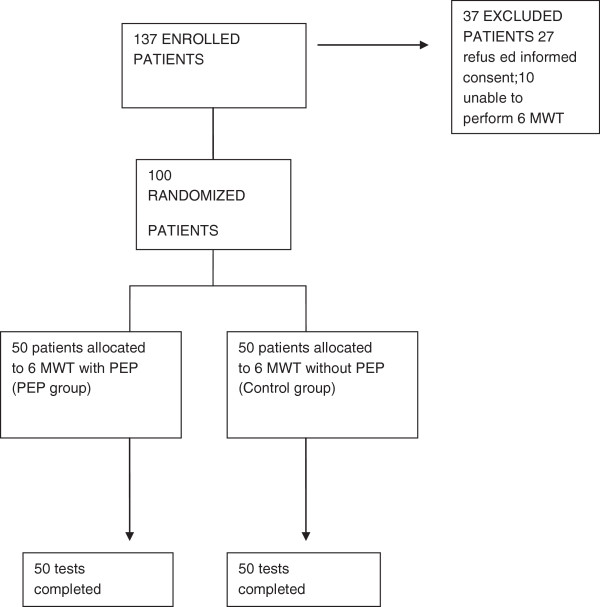
**Patients**’ **flow.**

137 patients were enrolled in the study and 100 were randomized (37 were excluded: 27 refused informed consent and 10 were unable to perform 6MWT). The demographic and baseline characteristics of the enrolled patients are reported in Table 1. The patients’ flow chart is reported in Figure 1. Pulmonary function testing was performed with a computerized body plethysmograph (VMAX 20 PFT Sensor Medics, Yorba Linda, CA,US), according to the international standards [14].

**Table 1:** **Patients**’ **characteristics at baseline**

	**PEP Group n** = **50 patients**	**Control group n** = **50 patients**
GENDER F/M	24/50	18/50
AGE years	71.9 ± 4.0	72.1 ± 4.1
FVC%	51.26 ± 11.89	49.67 ± 12.68
FEV1%	35.14 ± 14.56	33.48 ± 10.57
FEV1/FVC%	48.2 ± 8.4	53.2 ± 5.5
TLC%	147.36 ± 44.29	129.44 ± 10.27
RV%	148.82 ± 13.4	140.88 ± .13.22
DLCO%	54.68 ± 11.40	60.03 ± 0.10
paO2	67.1 ± 6.6	61.8 ± 7.0
paCO2	44.7 ± 3,3	43.8 ± 5.0
pH	7.41 ± 0,1	7.42 ± 0,2

DL_CO_, Lung diffusing capacity for carbon monoxide; FEV_1,_ Forced expiratory volume 1 sec; FEV_1_/FVC Tiffeneau index; FVC, Forced vital capacity; PaCO_2_, carbon dioxide arterial pressure. PaO_2_, oxygen arterial pressure; PEP, Positive Expiratory Pressure;RV, Residual volume; TLC, Total lung capacity.

**Figure 2:**
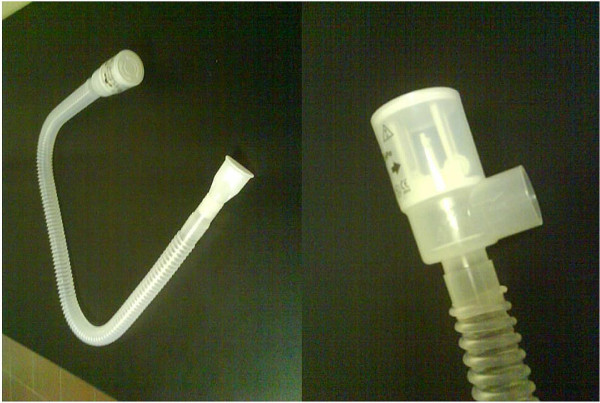
**Positive expiratory pressure (PEP) device used for 6 MWT composed by a 5 cm H2O Respironics PEP**-**valve**,**a tube and a mouthpiece.**

**Figure 3:**
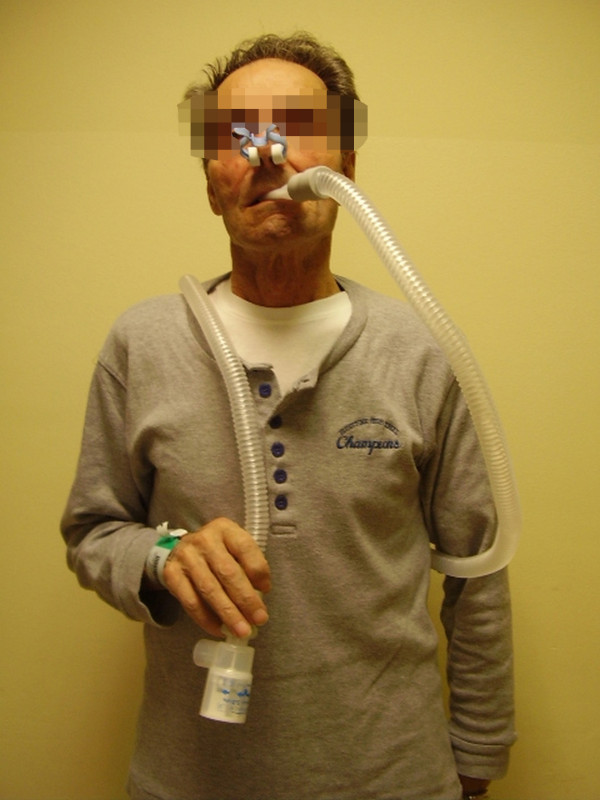
A participant using PEP device before 6 MWT.

Each patient underwent 6MWT in the first day, the test was performed twice to avoid learning effect [15]; the test of the two (performed the same day and separated each other by at least 30 minutes) with the longest covered distance was inserted in data analysis [15-17]. The following day the subjects were randomized to the PEP arm (50 patients) or to the Control arm (50 patients). PEP group patients repeated 6MWT only once using a PEP device consisting in a Respironics Threshold PEP valve set at 5cmH_2_O [18] connected to a 20 mm inner diameter and 100 mm tube and a mouthpiece (Figures 2 and 3) [19]. Control group patients repeated 6MWT only once breathing without this device. A different chest physiotherapist performed the 6MWT and he/she was blinded about the results of the previous test. 6MWT was performed according to the American Thoracic Society guidelines [20].

Oxygen saturation, heart rate, respiratory rate, dyspnea (using the Borg scale), and distance walked (meters) were recorded at the beginning and the end of every test as previously indicated [13]. A lightweight bluetooth wireless oxymeterNonin Avant 4000 was used for continuous oxygen saturation and heart rate measurements.

### Data analysis and statistics

Primary outcome was the difference in distance (meters) walked between the test before enrollment and the test after enrollment. Secondary outcomes were the differences in oxygen saturation, the reduction in dyspnea (Borg scale), and in respiratory rate. Descriptive data for continuous variables with a normal distribution are presented as mean ± standard deviation. Covariance analysis was used for assessing the difference between 6MWT with or without PEP. Multivariate analysis was used to evaluate correlations between cardio-pulmonary function parameters and patient performance. The effects of possible confounding factors (sex, age, meters walked on the first test) were determined by introducing these variables in the final model and calculating the change in the predictive factors coefficients.

Data analysis was done with statistics software R-Project version 7.13.2. A significance level of p < 0.05 was used for all comparisons.

## Results

All one hundred patients concluded the study. There were twenty-four females in the PEP group and eighteen in the control group. The average age of the participants was 71.9 ± 4.0 in the PEP group and 72.1 ± 4.1 in control group. 36 patients of the PEP group were being treated with association of inhaled β_2_ agonist plus corticosteroid and tiotropium bromide; the remaining 14 with β_2_ agonist and tiotropium. 39 patients of the PEP group were being treated with association of β_2_ agonist plus corticosteroid and tiotropium bromide; the remaining 11 with β_2_ agonist and tiotropium bromide. 3 patients of the PEP group and 4 patients of the control group had chronic respiratory insufficiency treated with oxygen.

All patients had similar reduction in lung volumes (FVC % 51.26 ± 11.89 in PEP group and 49.67 ± 12.68 in control group, FEV_1_% 35.14 ± 14.56 in PEP group and 33.48 ± 10.57 in control group). The 6MWT at baseline was 232.56 ± 101.59 in PEP group and 262.44 ± 95.02 in control group.

### Primary outcomes

**Figure 4:**
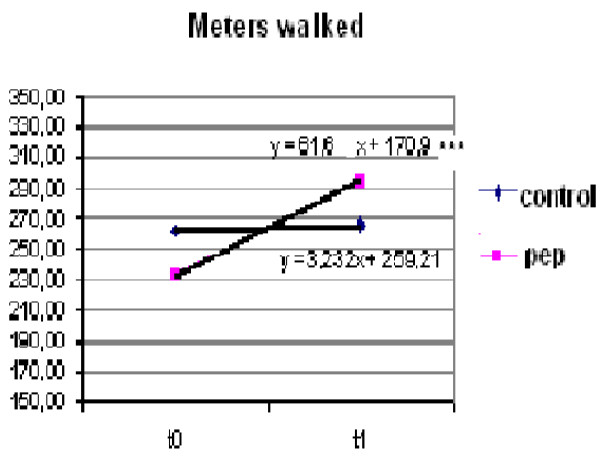
Trend analysis of distance (T0–T1) (meters walked) in the PEP group and in the Control group (p < 0.001).

Functional capacity assessed by the distance covered during 6 MWT improved in PEP group more than in the control group. The PEP group showed an increase in meters walked (61.66 ± 4.28) versus Control group (3.23 ± 0.59). The difference was statistically significant (p < 0.001). The mean values of distances walked and p-values are reported in Figure 4.

### Secondary outcomes

Oxygen saturation improved to a statistically significant level during 6 MWT (p < 0.01). Heart rate was reduced (p < 0.03). Also dyspnea (Borg scale) and the respiratory rate showed a reduction in the PEP group, but these did not achieve statistical significance. All results are showed in Table 2. The patients showing ability to maintain an adequate level of saturation improved the distance walked (multivariate analysis) (p < 0.03).

**Table 2:** Results of parameters evaluated before and after 6MWT in the two groups of patients (PEP group–Control group)

	**6MWT 1**^st^**TEST**		**6MWT 2**^nd^**TEST**		
	**(before randomization)**		**(after randomization)**		
	Group A (PEP)	Group B	Group A (PEP)	Group B	*p*
**Distance walked (meters)**					
	232.56 ± 101.59	262.44 ± 95.02	294.22 ± 97.31	265.67 ± 94.43	< 0.02
**Dyspnea (Borg)**					
Time 0*	1.63 ± 1.20	1.08 ± 1.03	1.49 ± 0.90	0.86 ± 0.97	0.4339
Time 1**	4.29 ± 1.75	4.22 ± 2.01	3.18 ± 1.65	4.41 ± 2.14	
**Respiratory rate**					
Time 0*	19.92 ± 5.26	20.68 ± 4.80	19.62 ± 4.64	20.65 ± 4.45	0.4884
Time 1**	26.68 ± 4.04	27.08 ± 4.35	23.38 ± 5.09	27.22 ± 3.28	
**Heart rate**					
Time 0*	80.17 ± 14.70	78.75 ± 10.23	80.17 ± 12.72	76.67 ± 13.48	<0.03
Time 1**	96.66 ± 15.76	97.36 ± 11.71	92.56 ± 12.57	98.96 ± 13.86	
**Oxygen saturation %**					
Time 0*	94.74 ± 0.01	94.61 ± 0.01	94.96 ± 0.02	94.84 ± 0.01	<0.01
Time 1**	88.97 ± 0.04	90.27 ± 0.04	90.75 ± 0.03	89.76 ± 0.04	

* At the beginning of the test.

** At the end of the test.

## Discussion

The present study suggests that adding a low positive pressure (5 cmH_2_O) PEP device enhances exercise capacity expressed as distance walked during 6MWT in patients with moderate to severe COPD. The principal effect of PEP device consists in increasing expiratory flow and decreasing pulmonary hyperinflation during exercise.

Expiratory flow limitation, which is the primary pathophysiological hallmark of chronic obstructive, is caused by reduced lung elastic recoil and increased airway resistance. Forced expiration associated with increased ventilatory demands during exercise can induce premature airway closure leading to air trapping and dynamic hyperinflation [13]. Dynamic hyperinflation contributes to increased elastic and mechanical loads on the inspiratory muscles and to neuroventilatory dissociation which further exacerbate the shortness of breath, leading to exercise intolerance, limited physical activity and thus to a poor quality of life [21]. Exercise tolerance is an important outcome measure in patients with COPD, because there is evidence that exercise testing is superior to other functional measurements obtained at rest in demonstrating the positive effect of a specific intervention. Some drugs such as formoterol and tiotropium demonstrate ability to change exercise tolerance and 6 MWT [22,23]. Non-invasive ventilation was used to improve exercise tolerance in COPD patients [24-27]. One of the various explored strategies to manage dynamic hyperinflation is to increase expiratory time as a result of slowing the respiratory rate by using low-level positive expiratory pressure [28]. Positive expiratory pressure devices can prolong expiratory time and decrease respiratory rate, thereby reducing airway closure and dynamic hyperinflation. The use of an expiratory positive airway pressure (associated with incentive spirometry) was described in a study in patients after coronary artery bypass graft. The application of a positive expiratory pressure improved the distance walked in 6MWT compared to controls [29]. The expiratory positive expiratory positive airway pressure promotes collateral ventilation, prevents airway collapse during expiration and thus reduces air trapping [29,30].

Another study about the use of an expiratory positive pressure device in COPD patients showed that the use of a conical-PEP produced an increase in inspiratory capacity of 200 ml, slowdown in vital capacity, and lung hyperinflation, and the improvement of the exercise endurance [13]. Martin and Davenport in a double blind crossover study showed that an extrinsic 10 cmH_2_O threshold PEEP reduced post-exercises dyspnea in COPD patients [9]. Finally, Monteiro and others published in 2012 a study similar to the our in which the demonstrated an increase in inspiratory capacity in patients with moderate to severe COPD treated with PEP applied trough oronasal mask after submaximal treadmill exercise [31].

However our study presents an important limitation: we did not measure the dynamic hyperinflation, and this is an important lack because a strict correlation exists between exercise dynamic hyperinflation, inspiratory capacity, dyspnea and exercise performance during 6MWT [32].

## Conclusions

Activity in COPD patients is limited by the development of dynamic hyperinflation. Changes in respiratory mechanics during exercise in patients with dynamic hyperinflation lead to exercise intolerance. The use of PEP during submaximal exercise may promote a reduction in the development of dynamic hyperinflation in COPD patients [31]. Our results has been obtained using only a low positive expiratory pressure (5 cm H_2_O) (the same obtained by pursed lips breathing) [13,18] without an inspiratory support or an inspiratory incentive device. In our opinion the strength of our study is the simplicity and the minor cost when compared to other devices and approaches (such as non-invasive ventilation or incentive spirometry with expiratory positive airway pressure or even conical-PEP). The use of PEP devices in rehabilitation programs is well known [9]: future studies would be needed to verify the usefulness of this device in the usual way (daily activity) during training and reconditioning.

### Consent

One of the participants of this study has given consent for publication of the image relating to him.

## Abbreviations

6MWT: Six minute walk test; COPD: Chronic obstructive pulmonary disease; PEP: Positive expiratory pressure; NIV: Non invasive ventilation; FVC: Forced vital capacity; FEV1: Forced expired volume 1 sec; CPT: Total pulmonary capacity; RV: Residual volume; CPAP: Continuous positive airway pressure; VT: Tidal volume; RR: Respiratory rate.

## Competing interests

The authors declare that they have no competing interests.

## Authors’ contributions

NA and MF designed the study, analyzed and interpreted the data, drafted and revised the manuscript. BC analyzed data collection and interpreted data, drafted and revised the manuscript. All authors have read and approved the final manuscript.

## References

[B1] Acquistapace F Piepoli M The walking test: use in clinical practice Monaldi Arch Chest Dis 2009 72 3 9 19645206 10.4081/monaldi.2009.336

[B2] Chetta A Pisi G Aiello M Tzani P Olivieri D The walking capacity assessment in the respiratory patient Respiration 2009 77 361 367 10.1159/000212781 19478551

[B3] Clini EM Crisafulli E Exercise capacity as a pulmonary rehabilitation outcome Respiration 2009 77 121 128 10.1159/000192773 19246958

[B4] Saetta M Finkenstein R Coslo MG Morphological and cellular basis for air flow limitation in smokers Eur Respir J 1994 7 1505 1515 10.1183/09031936.94.07081505 7957838

[B5] Carter R Holiday DB Stocks J Tiep B Peak physiologic responses to arm and leg ergometry in male and female patients with airflow obstruction Chest 2000 124 511 518 12907536

[B6] Killian KJ Leblanc P Martin DH Summers E Jones NL Campbell EJ Exercise capacity and ventilator, circulatory, and symptom limitation in patients with chronic airflow limitation Am Rev Respir Dis 1992 146 935 940 10.1164/ajrccm/146.4.935 1416421

[B7] Moga AM de Marchie M Saey D Spahija J Mechanisms of non-pharmacologic adjunct therapies used during exercise in COPD Respir Med 2012 106 614 626 10.1016/j.rmed.2012.01.006 22341681

[B8] Marin JM Carrizo SJ Gascon M Sanchez A Gallego B Celli BR Inspiratory capacity, dynamic hyperinflation, brethlessness, and exercise performance during the 6-minute walk test in chronic obstructive pulmonary disease Am J Respir Crit Care Med 2001 163 1395 1399 10.1164/ajrccm.163.6.2003172 11371407

[B9] Martin AD Davenport PW Extrinsic threshold PEEP reduces post-exercise dyspnea in COPD patients: a placebo-controlled double-blind cross-over study Cardiopulm Phys Ther J 2011 22 3 5 10 21886475 PMC3163412

[B10] Maltais F Reissman H Gottfried SB Pressure support reduces inspiratory effort and dyspnea during exercise in chronic airflow obstruction Am J Respir Crit Care Med 1995 151 1027 1033 7697226 10.1164/ajrccm/151.4.1027

[B11] Petrof BJ Calderini E Gottfried SB Effect of CPAP on respiratory effort and dyspnea during exercise in severe COPD J Appl Physiol 1990 69 79 88 10.1152/jappl.1990.69.1.179 2203722

[B12] Kyroussis D Polkey MI Hamnegard CH Mills GH Green M Moxham J Respiratory muscle activity in patients with COPD walking to exhaustion with and without pressure support Eur Respir J 2000 15 649 655 10.1034/j.1399-3003.2000.15d05.x 10780754

[B13] Padkao T Boonsawat W Chulee UJ Conical-PEP is safe, reduces lung hyperinflation and contributes to improved exercise endurance in patients with COPD:a randomized cross-over trial J Physiother 2010 56 33 39 10.1016/S1836-9553(10)70052-7 20500135

[B14] Miller MR Hankinson J Brusaco V Burgos F Casaburi R Coates A Crapo R Crapo R Enright P van der Grinten CP Gustafsson P Jensen R Johnson DC MacIntyre N McKay R Navajas D Pedersen OF Pellegrino R Viegi G Wanger J ATS/ERS Task Force Standardisation of spirometry Eur Respir J 2005 26 2 319 338 10.1183/09031936.05.00034805 16055882

[B15] Chadra D Wise RA Kulkarni HS Benzo RP Criner G Make B Slivka WA Ries AL Reilly JJ Martinez FJ Sciurba FC NETT Research Group Optimizing the 6-min walk test as a measure of exercise capacity Chest 2012 142 6 1545 1552 10.1378/chest.11-2702 23364913 PMC3515028

[B16] Hernandes NA Wouters EFM Meijer K Annegarn J Pitta F Spruit MA Reproducibility of 6-minute walking test in patients with COPD Eur Respir J 2011 38 261 267 10.1183/09031936.00142010 21177838

[B17] Casanova C Celli BR Barna P Casas A Cote C de Torres JP Jardim J Lopez MV Marin JM Montes de Oca M Pinto-Plata V Aguirre-Jaime A Six Minute Walk Distance Project (ALAT) The 6-min walk test distance in healthy subjects:reference standards from seven countries Eur Respir J 2011 37 150 156 10.1183/09031936.00194909 20525717

[B18] van der Schans CP de Jong W de Vries G Kaan WA Postma DS Koeter GH van der Mark TW Effects of positive expiratory pressure breathing during exercise in patients with COPD Chest 1995 105 782 789 8131541 10.1378/chest.105.3.782

[B19] Christensen EF Jensen RH Schonemann NK Petersen KD Flow-dependent properties of positive expiratory pressure devices Arch Chest Dis 1995 50 2 150 153 7613548

[B20] ATS statement Guidelines for six-minute walk test Am J Respir Crit Care Med 2002 166 111 117 12091180 10.1164/ajrccm.166.1.at1102

[B21] O’ Donnel DE Webb KA The major limitation to exercise performance in COPD is dynamic hyperinflation J Appl Physiol 2008 105 753 755 10.1152/japplphysiol.90336.2008b 18678624

[B22] Cazzola M Biscione GL Pasqua F Crigna G Appodia M Cardaci V Ferri L Use of 6-min and 12-min walking test for assessing the efficacy of formoterol in COPD Respir Med 2008 102 1425 1430 10.1016/j.rmed.2008.04.017 18621519

[B23] Nicolini A Short term effect of tiotropium on COPD patients treated with long acting bronchodilators Tanaffos 2012 11 1 26 31 25191397 PMC4153178

[B24] Menadue C Alison JA Piper AJ Flunt D Ellis ER Bilevel ventilation during exercise in acute on chronic respiratory failure: a preliminary study Respir Med 2010 104 219 227 10.1016/j.rmed.2009.08.015 19804963

[B25] Kohnlein T Schonheit-Kenn U WinterKamp S Weite T Kenn K Noninvasive ventilation in pulmonary rehabilitation of COPD patients Respir Med 2009 103 1329 1336 10.1016/j.rmed.2009.03.016 19362809

[B26] Menadue C Alison J Piper A Flunt D Ellis ER Non-invasive ventilation during arm exercise and ground walking in patients with chronic hypercapnic respiratory failure Respirology 2009 14 251 259 10.1111/j.1440-1843.2008.01449.x 19210652

[B27] Costes F Agresti A Court-Fortune I Roche F Vergnon JM Barthelemy JC Non invasive ventilation during exercise training improves exercise tolerance in patients with chronic obstructive pulmonary disease J Cardiopulm Rehabil 2003 23 4 307 313 10.1097/00008483-200307000-00008 12894005

[B28] Wouters EFM Non pharmacological modulation of dynamic hyperinflation Eur Respir Rev 2006 15 90 96 10.1183/09059180.00010007

[B29] Haeffener MP Ferreira GM MennaBarreto SS Arena R Dall’Ago P Incentive spirometry with expiratory positive airway pressure reduces pulmonary complications, improves pulmonary function and 6-minute walk distance in patients undergoing coronary artery bypass graft surgery Am J Heart 2008 156 900 e1-900. e8 10.1016/j.ahj.2008.08.006 19061704

[B30] Oliveira CC Carrascosa CR Borghi-Silva A Berton DC Queiroga F Jr Ferreira EM Nery LE Neder JA Influence of respiratory pressure support on hemodynamics and exercise tolerance in patients with COPD Eur J Appl Physiol 2010 109 681 689 10.1007/s00421-010-1408-8 20213467

[B31] Monteiro MB Berton DC Moreira FM Menna-Barreto SS ZimermannTeixera PJ Effects of expiratory positive airway pressure on dynamic hyperinflation during exercise in patients with COPD Respir Care 2012 57 9 1405 1412 10.4187/respcare.01481 22348429

[B32] Callens E Graba S Gillet-Juvin C Essalhi M Bidaud-Chevallier B Peiffer C Mahut B Delclaux C Measurement of dynamic hyperinflation after a 6-minute walk test in patients with COPD Chest 2009 136 1466 1472 10.1378/chest.09-0410 19581350

